# Modulation of airway hyperresponsiveness by rhinovirus exposure

**DOI:** 10.1186/s12931-018-0914-9

**Published:** 2018-10-29

**Authors:** Dennis Lo, Joshua L. Kennedy, Richard C. Kurten, Reynold A. Panettieri, Cynthia J. Koziol-White

**Affiliations:** 1 Department of Medicine, Rutgers Institute for Translational Medicine and Science, Rutgers University, New Brunswick, NJ USA; 2 Department of Pediatrics, Division of Allergy and Immunology, University of Arkansas for Medical Sciences, Little Rock, AR USA; 3 Department of Physiology and Biophysics, University of Arkansas for Medical Sciences, Little Rock, AR USA

**Keywords:** Rhinovirus, Airway Hyperresponsiveness, Airway smooth muscle, Inflammation

## Abstract

Rhinovirus (RV) exposure has been implicated in childhood development of wheeze evoking asthma and exacerbations of underlying airways disease. Studies such as the Copenhagen Prospective Studies on Asthma in Childhood (COPSAC) and Childhood Origins of ASThma (COAST) have identified RV as a pathogen inducing severe respiratory disease. RVs also modulate airway hyperresponsiveness (AHR), a key characteristic of such diseases. Although potential factors underlying mechanisms by which RV induces AHR have been postulated, the precise mechanisms of AHR following RV exposure remain elusive.

A challenge to RV-related research stems from inadequate models for study. While human models raise ethical concerns and are relatively difficult in terms of subject recruitment, murine models are limited by susceptibility of infection to the relatively uncommon minor group (RV-B) serotypes, strains that are generally associated with infrequent clinical respiratory virus infections. Although a transgenic mouse strain that has been developed has enhanced susceptibility for infection with the common major group (RV-A) serotypes, few studies have focused on RV in the context of allergic airways disease rather than understanding RV-induced AHR. Recently, the receptor for the virulent RV-C CDHR3, was identified, but a dearth of studies have examined RV-C-induced effects in humans.

Currently, the mechanisms by which RV infections modulate airway smooth muscle (ASM) shortening or excitation-contraction coupling remain elusive. Further, only one study has investigated the effects of RV on bronchodilatory mechanisms, with only speculation as to mechanisms underlying RV-mediated modulation of bronchoconstriction.

## Background

### In vitro studies - mechanisms of airway hyperresponsiveness

Airway hyperresponsiveness (AHR) is characterized by inflammation and bronchoconstriction in response to nonspecific stimuli [1] and is a key characteristic of chronic lung disorders such as asthma and chronic obstructive pulmonary disease (COPD). Exposure to environmental toxins has been an important area of study with respect to AHR. For instance, findings by Jude et al. [2] suggested that formaldehyde, an air pollutant, induced AHR in human airway smooth muscle (HASM) cells by altering contractile pathways in the cell. Ozone, another common pollutant, can induce AHR in human lung explants and ASM cells [3–5]. After toxicant exposure, cytokines liberated from airway structural cells or trafficking leukocytes modulate bronchomotor tone. Cooper et al. [6] demonstrated that human lung explants incubated with interleukin (IL)-13 exhibited AHR; other studies similarly found enhanced contractility after IL-13 exposure in HASM [7–9]. Studies by Amrani et al. [10] and Hotta et al. [11] have also implicated tumor necrosis factor-α (TNF-α) in the induction of enhanced ASM contractility. Respiratory pathogens have also gained attention as inducers of AHR. However, unlike for inflammatory cytokines, mechanisms whereby viral infection, specifically rhinovirus (RV), does this is subject to debate and largely have not been elucidated.

### Evidence of RV-induced AHR

Over the past few decades, RVs, the viruses precipitating the common cold, have been recognized as important pathogens in the development of wheeze leading to asthma and exacerbation of chronic respiratory disease. Evidence supports that RVs are the most prevalent respiratory viruses among patients with wheeze and asthma, especially in children [12–14]. Additionally, data from the Childhood Origins of ASThma (COAST) and Copenhagen Prospective Studies on Asthma in Childhood (COPSAC), which are high-risk birth cohort studies, established a link between RV-induced wheeze in children and risk/development of asthma [15, 16]. RV infections have also been implicated in exacerbations of asthma [17–20]. RV infection in vivo induces AHR, which is characterized by bronchoconstriction and inflammation of the airways [21–25]. Accordingly, investigators have pursued studies of AHR in non-diseased and asthma/COPD subjects after RV exposure.

### Studying molecular mechanisms underlying RV-induced AHR

Current models of RV-induced AHR include murine, single cell culture, cellular co-culture, and human models. Most studies have focused on in vitro systems to characterize inflammatory responses to RV. Infections of epithelial cell monolayers are useful for studying a single cell type, and epithelial cell co-cultures with other cell types have been successfully used to study the immune response [26]. However, even both approaches are incapable of capturing an integrated ex vivo model of cell-cell interaction critical to modeling infection and AHR that have been observed in vivo*.*

Murine studies provide a platform to study RV infections and AHR, but these studies are limited by species selectivity to minor group RV serotypes. Despite the development of a mouse strain expressing a chimeric intercellular adhesion molecule 1 (ICAM-1) for mouse recognition of RV-A16 [27, 28], what little experimental data exists is framed within the context of an allergic airways disease model rather than focusing on the direct effects of RV-A16 on AHR.

Obtaining approval for experimental RV studies in humans has been challenging, and disease context for asthma is limited to mild severity, leaving many questions left unanswered. Several prospective cross-sectional studies have shown associations between RV infection and asthma inception and exacerbations. However, these studies are limited in their ability to provide mechanisms that lead to AHR. Experimental challenge models in humans provide some opportunity to study AHR in the correct host and in the context of disease. In this review, we will discuss the current models and approaches to evaluate AHR during viral infection and speculate on potential mechanisms of RV-induced AHR as outlined in the known literature to date.

## Models of RV-induced AHR

### Animal models of RV infection

While human models of RV infection are most relevant, ethical concerns and practical limitations exist where investigators have chosen to use animal models. Yin and Lomax [29] developed a murine model of RV infection that mimics the effects of RV regarding inflammation, AHR, and exacerbations of allergic airways disease. Notable advantages to this model include an intact immune system, ability to genetically manipulate aspects of the immune response to determine the specific role in response to RV, and easy handling [30]. However, limitations lie in the inability to sustain RV titers after 24 h, as well as lack of responsiveness of murine ASM to a variety of bronchoconstrictors [31]. Additionally, many studies regarding acute RV-induced effects in vivo examine RV-B serotypes in mice since RV-A serotypes do not bind mouse ICAM-1 [27, 32] due to lack of homology with human ICAM-1. Studies on RV-B serotypes, however, are not may not be readily translational to human disease given that RV-A and RV-C serotypes have been associated with more severe symptoms in comparison to RV-B serotypes [33–36] and that many clinical studies of RV infection are proportionally skewed towards greater detection of RV-A and RV-C serotypes [20, 37–40].

In 2003, Tuthill et al. [28] developed an in vitro murine model suitable for major group RV (including RV-A16) infection. They achieved infection and replication of RV-A16 in a murine lower epithelial cell line expressing chimeric ICAM-1 with human domains for EC1 and 2. Similarly, Bartlett et al. [27] developed a BALB/c mouse strain transgenic for EC1 and 2 of human ICAM-1 that supports RV-A16 infection, thereby serving as a model that supports both RV-A and RV-B infection [41]. After viral infection, the transgenic mice exhibited replication-dependent features similar to those seen following RV-1B exposure, including airway inflammation, mucin production, and cytokine induction. Further, OVA-sensitized and challenged mice infected with RV-1B showed enhanced airway inflammation and hyper-responsiveness, serving as a murine model for RV-1B-induced allergic asthma exacerbations. A murine model for RV-induced COPD exacerbation was also developed by Singanayagam et al. [42]. Increases in TNF-α, interferon-γ-induced protein-10 (IP-10), and regulated on activation, normal T cell expressed and secreted (RANTES) expression were observed after a single elastase treatment followed by RV-1B infection. However, the translation of these models to human disease is tenuous.

While murine models are useful in elucidating some RV-induced effects in vivo, numerous immunologic differences exist between mice and humans that include differences in proportions of neutrophils and lymphocytes in peripheral blood and differences in Th_1_ differentiation [43]. Others have elegantly described the difference between murine and human lymphocytes: human lymphocytes demonstrate greater overlap in Th_1_ and Th_2_ cytokine profiles compared to murine lymphocytes [44]. Irvin and Bates [45] described anatomical differences between the murine and human lung, including those in lung branching pattern, airway caliber, thickness of epithelium, presence of submucosal glands, etc. Such factors could play a significant role in interpretation and extrapolation of results obtained from murine RV studies to RV studies in humans, making the findings of these studies challenging to translate to humans.

Recently, a cell culture system for RV-C serotypes was developed [46] and the receptor for RV-C serotypes, cadherin-related family member 3 (CDHR3), was identified [47]. The next step is exploring the pathogenic mechanism of RV-C infection in a murine model, possibly through transgenic mice expressing CDHR3. This new model may offer new opportunities in the study of virus-induced AHR.

### Airway cell co-culture models of RV infection

Studies have utilized an epithelial cell/airway smooth muscle co-culture model to examine cell-cell interactions in precipitating AHR. Damera et al. [48] found enhanced prostaglandin E_2_ in a co-culture of differentiated human airway epithelial and HASM cells after ozone exposure. Others found a 2.5-fold increase in HASM cell numbers in a co-culture with human airway epithelial cells (HAEC) compared to the HASM monolayer [49]. Although these single cell cultures are suitable for investigating secretion of inflammatory or contractile mediators, the monolayer model fails to reproduce the complex interactions of different airway cells in vivo. A co-culture cell model offers perhaps a more appropriate experimental design to study AHR.

Few studies have explored airway co-culture models to study RV infection (Table 1). However, the effects of RV-conditioned media on airway cells has been studied. Van Ly et al. [50] infected primary HAECs with RV for 24 h and observed β_2_ adrenoceptor (β_2_AR) desensitization in primary HASM after exposure to the RV-induced medium. Similarly, Rajan et al. [51] co-cultured human peripheral blood mononuclear cells (PBMCs) and RV-infected HAEC in a two-chamber trans-well tissue culture system and found increased levels of interferon (IFN) λ2 (also known as IL-28A), IFN-α, monocyte chemoattractant protein (MCP)-2, and macrophage inflammatory protein (MIP)-1β. The synergistic effects of the co-culture model showed elevated levels of IP-10 and MCP-1 in a primary HAEC/human monocyte co-culture after RV exposure [52]. Shariff et al. [53] identified that exposure of medium from RV-infected primary HAECs led to primary ASM chemotaxis, primarily modulated by RANTES. However, the number of studies using co-cultures to study airways disease has been limited due to the necessity of air-liquid interface culture as well as primary HAECs. A study by Pezzulo et al. [54] confirmed that the transcriptional profile of differentiated HAECs more closely resembles the human airway epithelium than transformed airway epithelial cell lines. Therefore, the use of primary epithelial cells in co-cultures rather than utilizing transformed epithelial cell lines is the most physiologically relevant system to study the effects of RV.

**Table 1:** Rhinovirus Studies

Method/Study	Advantages	Disadvantages	References
ASM cells	Primary cell modulating AHR and airway tone	Direct infection with RV not likely given architecture of the lung	Hakonarson 1998 [73]; Van Ly 2013 [50], Shariff 2017 [53];
Co-cultures of airway cells	Integrated response of multiple cell types	Few studies elucidating modulation of RV-induced AHR	Korpi-Steiner 2010 [52]; Van Ly 2013 [50]; Rajan 2013 [51]
Clinical isolates	Tissue from infected patients with and without asthma/COPD	Inconsistent findings with respect to susceptibility to infection/symptoms	Marin 2000 [94]; Corne 2002 [95]; Greene 2002; de Kluijver 2003 [108]; DeMore 2009 [109]; Schneider 2010 [96]; Kennedy 2014 [18]
PCLS	Intact architecture of the lung tissue/airways	No circulating immune cells	Kennedy and Koziol-White 2018 [24]
Murine studies	Easy to manipulate genetically to understand mechanisms of RV-induced AHR	Only susceptible to RV-B infection, a serotype not associated severe RV infections/symptoms. Model with human ICAM-1 limited to RV infection in the context of allergic airways disease.	Tuthill 2003 [28]; Bartlett 2008 [27]; Meurs 2008 [31]; Calvo 2009 [34]; Lau 2009 [35]; Miller 2009 [36]; Bizzintino 2011 [33]
Pediatric in vivo studies	• Correlation between RV exposure and wheeze in a large population of pediatric subjects. • Identification of potential targets for abrogation of RV-induced AHR/development of asthma. • Experimental RV challenge to study relationship between IgE levels and exacerbation severity.	• Primarily performed in subjects with European ethnic background. • Little extrapolation to other ethnic groups due to prevalency of polymorphism associated with wheeze.	Lemanske 2002 [16]; Kotaniemi-Syrjänen 2003 [80]; Zambrano 2003 [25]; Bisgaard 2004 [15]; Jackson 2008 [64]; Calişkan 2013 [89]

### Ex vivo tissue models of AHR and respiratory virus exposure

Primary airway cell culture and precision cut lung slices (PCLS) are currently used to study effects of a number of lung insults ex vivo (Table 1). While primary cell culture and in vitro co-culture consist of only a few cell types, human PCLS prepared from organ donors consist of many relevant cell types of the respiratory tract situated within their native micro-anatomical environment. Maintaining the in vivo properties of the cultured cells, such as structure, promotes PCLS as an especially suitable model for studying airways disease [26]. For instance, this system also maintains ciliary motility and responsiveness to contractile agonists, including carbachol (CCh), a cholinergic agonist much like methacholine. Kennedy et al. [24] assessed carbachol responsiveness after infection with RV39 in PCLS from donors with a history of asthma. Other viruses have also been studied using this approach. Sun et al. [55] studied the human respiratory syncytial virus (RSV) and found a response to RSV immunostimulatory defective viral genomes (iDVG) in PCLS. Avgousti et al. [56] found that protein VII, a highly basic cellular histone-like protein encoded by adenoviruses, alters cellular chromatin in human PCLS. Furthermore, Kim et al. [57] found that deficiency in melanoma differentiation-associated protein (MDA)-5, a protein that detects motifs in viral oligonucleotides, enhanced constriction in mruine PCLS after infection with Sendai virus. Another study showed incubation of human PCLS with high-concentration poly (I:C) had little effect on contractility but induced secretion of cytokines and chemokines [6].

Despite the relevance of the human PCLS in studying RV-induced AHR, the limited availability of human lung tissue makes animal PCLS a viable alternative. Ressmeyer et al. [58] studied bronchoconstriction in guinea pig PCLS and found similar responsiveness to endogenous mediators when compared to humans. Henjakovic et al. [59] reported that murine PCLS exposed to trimellitic anhydride and DNCB, two allergenic sensitizers, exhibited the same effects as observed in vivo*.* Furthermore, Fox [60] observed that porcine PCLS shared similar contractile agonist sensitivity with human airways and studied inflammatory mediator secretion by PCLS from the murine model of allergic airways disease.

### Clinical models of RV infection

A number of studies have investigated the clinical effects of RV exposure in human subjects in both cross-sectional studies and in human challenge models (Table 1). An early RV challenge study by Grunberg et al. [22] found a decrease in the provocation concentration for a 20% decrease in forced expiratory volume in 1 s (FEV_1_) and increases in IL-8 levels in atopic asthmatic subjects with RV-A16. A cross-sectional clinical study found increased AHR to methacholine in children (ages 7–12 with intermittent asthma) with RV-induced asthma during the course of reported colds [61]. These studies confirmed the findings of earlier work by Zambrano et al. [25], which showed increased methacholine sensitivity in highly atopic individuals (ages 18–30, total IgE > 371 IU/mL) during RV-A16 experimental challenge. Proud et al. [62] found significantly increased symptom scores in RV-infected subjects compared to sham-infected controls (in 20 year old subjects), and others have shown increased IL-25 and IL-33 in human subjects (ages 26–36, and ages birth to 6 years old) after experimental RV-A16 infection [63, 64]. Since majority of studies have investigated RV in the context of underlying airways disease, the fundamental mechanisms through which RV modulates AHR remains elusive.

There is evidence for neurogenic inflammation playing a part in both asthma and rhinitis, a hallmark of respiratory tract infections with virus. Such findings suggest that neurokinins and innervation of the airways may play a role in modulating RV-induced AHR via changing the responses of nerves in the airways [65, 66]. A single study of five subjects with colds noted that inhalation of substance P, which stimulates cough responses in guinea pigs following release from the nerves [67], induced cough in normal subjects infected with RV but had little effect on subjects without RV infection [68]. The therapeutic that was effective at diminishing these effects was procaterol, a β_2_ adrenergic receptor agonist, which mainly targets airway smooth muscle to induce bronchodilation of the airways. However, this study did not directly test the idea that RV infection induces release of neurokinins into the airways to induce AHR. Thus far, there have been no studies linking neurokinin receptor agonists like substance P to RV-induced AHR, only a putative role for receptors for these agents in respiratory syncytial virus infection in rats [69, 70]. A study by Abdullah et al. shows that RV upregulates transient receptor potential (TRP) channels, which are known to modulate neurogenic signals and have been implicated in cough [71], in neurons. To date no other studies have been performed to expand upon this study, so it is unclear as to whether TRP channels play a role in RV-induced AHR. Additionally, an experimental RV infection model in human subjects noted that RV infection enhanced responsiveness to histamine rather than bradykinin, thereby suggesting that airway sensory nerves may not play much of a role in RV-induced asthma exacerbations [22]. The findings of all of these studies that suggest a role for neurogenic inflammation in RV-induced AHR are difficult to use to establish clinical relevance for the paradigm because: (1) there is significant interspecies variation between human and animal models, and (2) there is a significant amount of afferent sensory innervation variability among human subjects [72].

## Understanding mechanisms of RV-induced AHR and ASM cell function

Currently, there is limited understanding of mechanisms by which RV modulates airway contractility. Given that ASM is the pivotal cell modulating bronchomotor tone and hyperreactivity, modulation of contraction and relaxation of this tissue is a logical starting point to discover mechanisms of RV-induced AHR. Upon finding that RV enhanced airway contractile responsiveness and reduced β-agonist isoproterenol efficacy in isolated rabbit and human ASM, Hakonarson et al. [73] found diminished cAMP levels, increased G_αi_ expression, and enhanced ICAM-1 expression on the cell surface of ASM. While an increase in ICAM-1 density on the cell surface can enhance RV binding to improve infection rates, higher G_αi_ expression and lower cAMP level could then contribute to RV-induced airway hyperresponsiveness. As G_αi_ inhibits adenylyl cyclase, cAMP levels diminish and subsequently, Protein Kinase A (PKA) fails to inactivate myosin light chain kinase (MLCK) to induce bronchodilation. According to McGraw et al. [7], increased G_αi_ decreased β_2_AR-mediated bronchodilation and M_3_-muscarinic receptor (M_3_R)-mediated bronchoconstriction in murine ASM. Interestingly, this finding translates to a protective effect with regard to bronchoconstriction in conjunction with reduced effect of ß_2_AR-agonists. Others postulated that G_αi_ upregulation activates Rho via Rho guanine nucleotide exchange factors (GEFs), that then evokes actin polymerization in ASM (as described in [74–77]). Croxton et al. [78] found that modulation of G_αi_ contributes to Ca^2+^ sensitization in tracheal smooth muscle, thereby potentially increasing airway contractility.

### RV-induced effects on ASM excitation-contraction (E-C) coupling

To date, few studies have been conducted examining mechanisms of RV-induced modulation of airway contractility. Hakonarson et al. [79] identified IL-1β to be involved in rabbit and HASM responsiveness following RV exposure. After pretreatment of rabbit tissue with an IL-1 recombinant human receptor antagonist, RV-induced changes in responsiveness were attenuated. Additionally, RV exposure induced IL-1β expression, which was ICAM-1 dependent. According to Van Ly et al. [50], HAECs exposed to RV secreted factors into the media that induced β_2_AR desensitization in human ASM through TLR activation of cyclooxygenase-2-induced prostaglandins. Kennedy et al. [24] found that RV39 exposure of non-asthma-derived human precision cut lung slices failed to induce hyperresponsiveness to a contractile agonist, but exposure of asthma-derived lung slices induced hyperresponsiveness of the small airways. Overall some studies suggest potential mechanisms underlying RV-induced AHR; however, more work is needed to thoroughly examine mechanisms of this phenomenon.

## Clinical studies: RV and the development of wheeze and AHR

Rhinoviruses have been implicated in the development of wheeze that evokes childhood asthma. Johnston et al. [13] identified RVs induce two thirds of upper respiratory viral infections in children (ages 9–11) with cough/wheeze through a community based longitudinal study. Similarly, RVs were detected in 33% of nasopharyngeal aspirates in children hospitalized for wheezing. In the same study, RV-positive cases were associated with development of asthma (OR = 4.14) while RV-negative cases were not [80]. Notably, it was reported that ~ 90% of children with RV wheezing illness in the third year had developed asthma by the sixth year [64]. The COAST birth cohort study revealed a significant role of RVs in pediatric wheezing illness and development of asthma [81–84]. RV was associated with asthma at 13 years as well as significantly reduced lung function measures compared to measures observed with exposure to other respiratory viruses. In another high-risk cohort study, a statistically significant association between wheezy lower respiratory infection by RV and current asthma, persistent wheeze, and current wheeze was reported [85]. Recently, findings from a meta-analysis confirmed a possible association between RV-induced wheeze in the first 3 years and development of wheeze or asthma [86]. Other factors may co-influence the association between RV and the inception of asthma. Lukkarinen and colleagues studied 127 steroid-naive children with a first episode of wheeze [87]. Subjects were enrolled around 11 months of age and re-evaluated for asthma at age 7 years. The authors found that 37 of these children maintained an asthma diagnosis and were atopic at the follow-up. The risk factors discovered at study enrollment for future atopic asthma included allergic sensitization (OR 12; *p* < 0.001), eczema (OR 4.8; *p* = 0.014), and wheezing with rhinovirus (OR 5.0; *p* = 0.035). Although the above studies provide strong evidence for a relationship between RV and the development of wheeze/asthma, it was not possible to determine whether RV-induced wheezing precipitates asthma. While the causation relationship is a possibility, another plausible hypothesis is that RV-induced wheeze in infancy is only a sign of predisposition to development of asthma.

### Atopy, RV, and wheezing

Atopy is a risk factor that has been implicated in the development of RV-induced exacerbations of asthma. A positive association between IgE sensitization and RV wheezing illness in hospitalized wheezing children was found [88]. In another study, RV-infected children sensitized to allergens exhibited higher odds of wheezing compared to controls [20]. A genetic predisposition toward developing wheeze leading to asthma has also been impugned. A susceptibility locus on the 17q21 chromosome in COAST and COPSAC cohorts was discovered [89]. The 17q21 genotypes were found to have significant association with children with RV wheezing illness but not with those without RV wheezing illness. Interestingly, previous studies (genome-wide association studies in particular) have identified the locus in the 17q21 chromosome to be linked to the development of asthma [90, 91]. However, since the birth cohort study encompassed subjects of primarily European descent, the findings may not be applicable to the general population. Bochkov et al. [47] similarly identified an asthma susceptibility gene product in CDHR3 and proposed that the Tyr_529_ variant at rs6967330 (rs6967330-A) in the *CDHR3* gene is a potential risk factor for RV-C wheezing illness. The COPSAC_2010_ and COAST birth cohorts confirmed the association of the rs6967330-A variant with respiratory episodes with RV-C detection [92]. The 1000 Genomes Projects found rs6967330-A to be most predominant in African populations [92], which report high asthma incidence and morbidity [93].

## RV-induced AHR in asthma, COPD, and healthy subjects

RVs have been found to play an important role in the context of chronic respiratory disease. Studies have demonstrated that although healthy patients respond to RV with respiratory symptoms, those with an underlying airways disorder respond to a greater degree when exposed to the same virus/amount of virus. RV RNA was detected in nasopharyngeal swabs of 32.4% of asymptomatic asthmatic children compared to none in controls, implying the persistent presence of RV in asthmatics [94]. Others found that subjects with asthma were not more susceptible to RV infection than controls but exhibited more severe and longer-lasting lower respiratory symptoms [95]. Similarly, elevated baseline cytokine levels in primary COPD-derived epithelial cells appeared to increase susceptibility of COPD epithelial cells to RV infection due to significantly increased viral titer and copy number post infection [96]. Thus, an important area of focus examines the effects of RV infection in normal compared to diseased subjects due to the role of RVs in the exacerbation of respiratory symptoms.

One key feature observed in RV-infected asthma subjects is the possibility of an impaired innate immune response in the host. In vitro studies by Wark et al. [97] found that apoptotic responses were impaired and IFN-β secretion was > 2.5 times lower in primary HAECs from asthmatic subjects compared to healthy controls. This clearly demonstrated the compromised host innate immune response in asthma and susceptibility to viral replication. Asthma subjects also exhibited eosinophilic inflammation in addition to significant reduction in FEV_1_, peak expiratory flow, provocative concentration of histamine, and total blood lymphocytes after RV infection [98]. Such changes were associated with reduced production of Th_1_ cytokines IFN-γ and IL-10 as well as increased production of Th_2_ cytokines IL-4, IL-5, and IL-13. Similarly, Sykes et al. [99] determined significantly diminished concentrations of type I interferon proteins IFN-α, IFN-α2, and IFN-β in BAL cells of RV-infected asthmatic subjects. Additionally, deficient type I interferon production was associated with AHR and more positive skin prick test responses, and was associated with more severe childhood asthma [100–102]. Baraldo et al. [103] and Pritchard et al. [104] have also found diminished levels of type I interferons after RV infection in bronchial biopsies from asthmatic children and from media in which PBMCs from asthmatic subjects were cultured, respectively. It is important to mention that others have seen similar levels of type I interferons and IL-15 in asthmatics during RV-induced exacerbations of asthma [18, 105], suggesting that innate immune deficiency may only be relevant in specific patient subtypes.

A notable factor contributing to severity of RV-induced AHR is atopy, although there are conflicting findings. In a study of allergic and non-allergic subjects, Gern et al. [106] found that RV infection induced an increase in airway responsiveness to histamine in allergic subjects and no change in controls. Others found that allergic children experienced more upper respiratory symptoms and asthma exacerbations as well as prolonged duration of AHR compared to controls [61]. Furthermore, in a case-control study the combination of sensitization and exposure to allergen and detection of respiratory virus heightened the risk of hospitalization for asthma [107]. However, chronic low-dose house dust mite allergen exposure did not exert a synergistic effect with RV infection in mild to moderate allergic asthma [108]. Mild allergic subjects with asthma did not exhibit more severe symptoms compared to healthy subjects [109]. Interestingly, treatment with CNTO3157, an inhibitory monoclonal antibody to Toll-like receptor 3 (TLR3), failed to prevent an RV-induced fall in FEV_1_ and airway symptoms in subjects with asthma but ameliorated symptoms in healthy subjects [110]. This finding implies that inflammation may be unchecked in asthma due to viral infection. Additionally, the use of non-specific anti-inflammatories like corticosteriods has been limited in its efficacy in certain subsets of RV-infected individuals. This is supported by evidence demonstrating that inhaled corticosteroids provide limited protection against virus-induced asthma exacerbations [111, 112].

## Rhinovirus in the context of air pollution

Several studies have examined the effects of air pollutants on airway reactivity and inflammation, as well as the effects of cigarette smoke. However, there has been a paucity of studies examining the effects of air pollutants on RV-induced inflammation or AHR, or how RV infection impacts the effects of air pollutant exposure. Many of the studies have been in vitro studies of single cell types. Some studies of cigarette smoke (CS) exposure in the context of RV in airway epithelial cells note that RV replication is increased following CS exposure [113, 114], but others have found that there was little effect on viral titer [115, 116]. CS exposure has also been found to modulate RV-induced gene expression and inflammatory mediator release from airway cells, decreasing innate inflammatory gene expression and release of IP-10 [113–116] while increasing release of IL-8 from epithelial cells [117]. Capistrano et al. noted that exposure to biomass smoke had no effect on viral load, but enhanced RV-A16-induced IL-8 production but not IL-6 production from human lung fibroblasts [118]. Oxidant pollutant exposure, particularly NO_2_ and O_3_, alone can induce release of inflammatory mediators from airway epithelial cells. When combined with RV-A16 it was noted that IL-8 release from BEAS-2B cells was significantly augmented over RV-A16, NO_2_, or O_3_ alone [119]. However, a study of experimental RV exposure with exposure to O_3_ following virus inoculation noted that there were no differences in the following parameters: RV titers, recruitment of neutrophils, interferon levels in nasal lavage, or changes in serum neutralizing antibody to RV [120]. The disconnect between the aforementioned studies could be due the different parameters that were studied, or could be due to an in vitro versus an in vivo study.

Exposure to particulate matter (PM2.5) alone induces wheezing and inflammation in individuals exposed to it. An observational study noted that there was a significant interaction between PM2.5 and RV infection in asthmatics that caused a significant decrease in both FEF_25–75_ and FEV1% predicted values compared to asthmatics with no RV infection [121]. Interestingly, a study in rat lung epithelial cells noted that exposure to diesel exhaust particles upregulated expression of ICAM-1 and LDL, both receptors for different RV serotypes [122], suggesting that exposure to diesel exhaust particles may serve to enhance sensitivity of the airways to RV infection. These findings suggest that the effect of air pollution on RV-induced AHR and inflammation appear to be dependent on the type of pollution that the individual is exposed to, and whether or not the individual has underlying lung disease. Overall, there seems to be an additive effect of air pollution on top of what RV infection already elicits.

## Summary

Although significant work has been performed studying the effects of RV exposure/infection on modulation of lung inflammation alone, or in the context of asthma and COPD, molecular mechanisms underlying RV-induced AHR remain unclear. Additionally, whether alterations in these mechanisms in subjects with asthma or COPD is what drives more severe AHR and respiratory symptoms is also uncertain. New and novel models of the lung will provide insight into targets to abrogate RV-induced AHR in ASM, as well as to abrogate RV-induced exacerbations of underlying airways disease.

The use of antivirals in treatment of RV infection clinically has failed, and it is unlikely that a targeted RV therapy will be developed due to the large diversity of serotypes. Targeting structural cells of the airways presents a unique approach to abrogate RV effects on AHR and inflammation. Identification of inflammatory mediators inducing sustained AHR and novel pathways involved in alterations in contractility of the airways may serve as targets for therapeutics in subjects both with and without lung disease. Traditional therapy for RV-induced exacerbations, mainly the use of corticosteroids, has been shown to be ineffective in the treatment of RV-induced AHR and inflammation. Conversion of an exacerbation of asthma to nothing more than a common could would be a new paradigm that would decrease asthma morbidity and mortality. A more focused approach targeting soluble mediators that enhance excitation-contraction coupling in airway smooth muscle may provide a more precise approach in treatment of RV-induced exacerbations of asthma.

## Abbreviations

AHR: Airway Hyperresponsiveness
ASM: Airway Smooth Muscle
cAMP: Cyclic Adenosine Monophosphate
Cch: Carbachol
CDHR3: Cadherin-related family member 3
COAST: Childhood Origins of ASThma
COPD: Chronic Obstructive Pulmonary Disease
COPSAC: Copenhagen Prospective Studies on Asthma in Childhood
E-C: Excitation-Contraction
FEV_1_: Forced Expiratory Volume in 1 s
GEF: Guanine nucleotide Exchange Factors
HAEC: Human Airway Epithelial Cells
ICAM-1: Intracellular Adhesion Molecule 1
iDVG: Immunostimulatory Defective Viral Genomes
IFN: Interferon
IgE: Immunoglobulin E
IL: Interleukin
IP-10: Interferon gamma-induced protein 10
M_3_R: Muscarinic Receptor 3
MCP: Monocyte Chemoattractant Protein
MIP: Macrophage Inhibitory Protein
OR: Odds Ratio
OVA: Ovalbumin
PBMC: Peripheral Blood Mononuclear Cells
PCLS: Precision Cut Lung Slices
RANTES: Regulated on activation, normal T cell expressed and secreted
RNA: Ribonucleic Acid
RSV: Respiratory Syncytial Virus
RV: Rhinovirus
Th: T helper cell
TNFα: Tumor Necrosis Factor α
TRP: Transient receptor potential
Tyr: Tyrosine
β_2_AR: Beta 2 Adrenergic Receptor
